# Orthorectic behavior among students and motivation for physical activity, dietary habits, and restrictive eating

**DOI:** 10.3389/fnut.2024.1367767

**Published:** 2024-06-03

**Authors:** Mateusz Rozmiarek, Mateusz Grajek, Karolina Krupa-Kotara, Ewa Malchrowicz-Mośko

**Affiliations:** ^1^Department of Sports Tourism, Faculty of Physical Culture Sciences, Poznan University of Physical Education, Poznan, Poland; ^2^Department of Public Health, Faculty of Public Health in Bytom, Medical University of Silesia in Katowice, Bytom, Poland; ^3^Department of Epidemiology, Faculty of Public Health in Bytom, Medical University of Silesia in Katowice, Bytom, Poland

**Keywords:** orthorexia, dietary habits, restrictive eating, physical activity, students, motivation

## Abstract

**Introduction:**

Orthorexia lacks official recognition as an eating disorder; however, orthorexic behaviors, associated with a stringent selection of food, may serve as a predisposing factor to the development of eating disorders. It is characterized by an obsessive preoccupation with healthy eating and strict dietary standards, often prevalent in high-risk groups such as athletes and individuals concerned with their physical appearance. The objective of this study was to evaluate the incidence of orthorexia among students exhibiting varying lifestyles (including dietary habits and levels of physical activity, along with their respective motivational factors). The research involved 600 participants equally distributed between health-related (HRF) and non-health-related (NRF) academic categories, with the majority of participants in the HRF category being women.

**Methods:**

Assessments included BMI calculations, dietary evaluation based on Polish standards, categorization of physical activity levels, the EMI-2 questionnaire on motivation to exercise, the DOS test for orthorexia propensity, and the TFEQ-13 questionnaire for eating behavior.

**Results and discussion:**

Results showed that primary motivators for physical activity included disease prevention, health maintenance, strength/endurance, and physical appearance. Orthorexia tendencies were prevalent, particularly in the HRF group, linked to lower BMI, better diet quality, higher physical activity levels, and a higher prevalence of restrictive eating. In conclusion, students in health-related fields, driven by a strong health consciousness, are at risk of orthorexia. This emphasizes the need for balanced health education and support.

**Conclusion:**

Orthorexic tendencies are associated with distorted perceptions of food portions and calories, underscoring the importance of awareness and intervention.

## 1 Introduction

Recently, there has been a growing interest in physical activity and diet, as well as the pursuit of an ideal figure (1). In some cases, attention to health and image takes the form of obsession (2). This justifies the need to research the prevalence of eating disorders. People affected by them hold incorrect beliefs about nutrition, especially regarding energy provision and weight gain (3). Consequently, great emphasis should be placed precisely on assessing the risk of eating disorders in different population groups, with a particular focus on young and health-intensive individuals (4, 5). This is supported by the observation that the prevalence of eating behaviors that have features of eating disorders with atypical symptoms has increased in recent years (6). To distinguish this group, they are called nonspecific eating disorders (7, 8), which is also justified by the fact that they are searched for in vain in the official classifications of mental disorders (DSM-V), and in the Statistical Classification of Diseases (ICD-10) they have codes F50.8 and F50.9—other eating disorders; eating disorders, unspecified (in ICD-11 the entry is expanded to eating and eating disorders nowhere specified) (9–11).

One of the non-specific/unspecified eating disorders is orthorexia (12). The term was first used in 1997 by Bratman, indicating the possible emergence of a novel eating disorder, delineated as follows: “a pathological focus on healthy eating with features of rigorous attention to food quality and preparation and dietary norms” (13)—for this reason, orthorexia is sometimes classified as an obsessive-compulsive disorder (also known by the acronym OCD). According to Bratman et al. (6), this dietary pattern may be classified as a mental disorder on account of its physiological, psychological, and societal repercussions. Importantly, orthorexia can be multidimensional and encompass several neurobiologically-based variants (14, 15). The treatment of orthorexia in the category of an eating disorder is because the orthorexic loses objectivity about a healthy lifestyle, and how to eat and engage in physical activity. The given behaviors are performed schematically, and deviating from them triggers negative emotions. Symptoms of orthorexia involve making choices that are socially recognized as health-promoting, but in an exaggerated and radical way (16). It is estimated that orthorexia may occur in 1–7% of the general population (depending on the measurement methodology adopted) (12, 17–20), and in high-risk groups (athletes, dieters, and appearance-conscious individuals) this risk can increase to 60% and even 90% (7, 20). What is most interesting about orthorexia is that it is a kind of alternative to other eating disorders (6). From a psychopathological point of view, it is not clear whether orthorexia should be categorized as an eating disorder or as OCD. Considering the eating symptoms, it can be considered a non-specific eating disorder (21, 22). Given the cognitive-behavioral framework, it aligns more closely with obsessive-compulsive disorder (23, 24). The diagnostic criteria for orthorexia proposed in the literature define it as an independent pathological entity, not obligatorily including such features as body anxiety and general dissatisfaction with body weight (25), as well as a sense of detachment from one’s own body (6, 26). In the course of the disorder, a person feels a sense of control over his or her own life due to maintaining a strict diet. The orthorexic’s diet revolves around the quality of meals and the search for better and better food and the obvious inability to meet his or her demands leads the orthorexic to nutrient deficits (27, 28).

An individual diagnosed with orthorexia exhibits distinctions from someone diagnosed with a specific eating disorder (note: anorexia, bulimia, and BED) in that the former does not possess an obsessive drive to attain a slender physique. Instead, any deviation from the planned diet or the perception of a nutritional misstep is met with unwarranted health-related apprehension. Conversely, there is a fixation on meticulously monitoring the nutritional content of meals consumed, accompanied by a fear of ingesting anything deemed unhealthy, which leads to an avoidance of foods with un-disclosed composition or origin (7, 23, 24). Other consequences of orthorexia include the elimination of foods on the menu that do not meet the standards imposed by the orthorexic, as well as the avoidance of social contacts that would pose a risk of eating foods with unknown composition. As a result, the life of a person suffering from orthorexia revolves around searching for food, making food choices, and planning diet and physical activity (27, 28).

Commonly acknowledged predisposing factors implicated in susceptibility to the development and perpetuation of eating disorders, including orthorexia, encompass body image, perfectionism, attachment style, and self-esteem (29). These predictors were assessed in a study by Barnes and Caltabiano (30). The researchers discovered that elevated orthorectic tendencies exhibited a notable correlation with heightened scores on self-oriented perfectionism, appraisal orientation, and anxious and rejecting attachment style. Excessive fixation on weight, appearance orientation, and a history of eating disorder were identified as substantial predictors of orthorexia. Similar results were obtained by Bardone-Cone et al. (31) and Brown et al. (32).

In addition, researchers emphasize that in the case of orthorexia, an additional predisposing factor is a lifestyle and social role (e.g., occupation related to esthetic appearance) (28, 33, 34). The latter is confirmed by, among others, Asil and Sürücüoğlu (35), who found that about 50% of dieters are at risk for orthorexia, and 12.9% for other eating disorders. Another study involving health science students found orthorexic behavior in 68.2% of respondents (36). Similar results were found among those characterized by high physical activity and attention to body shape (26, 37). The results of the study showed that as many as 45.5% of medical students exhibited characteristics of orthorexia, while 88.2% of surveyed dietitians and 52% of surveyed physical therapists declared a change in their approach to food and nutrition after entering medical school (38).

Another study found a weak relationship between studying dietetics and the incidence of eating disorders (39). Arslantas et al. (40) demonstrated that nurses who prioritized sound nutrition and expressed apprehension regarding weight gain were at an elevated risk of developing orthorexia. Almeida et al. (41), on the other hand, showed an association between orthorexic tendencies and frequent exercise and dis-satisfaction with body image.

In light of the aforementioned relationships, it has been decided to conduct a study aimed at a thorough assessment of the prevalence of orthorexia among groups of students characterized by diverse lifestyles, encompassing both dietary habits and physical activity, including their motivation to engage in these behaviors. The research hypotheses were constructed: (1) The motivating factors for physical activity in the study sample are health issues; (2) The occurrence of orthorexia is associated with a normal BMI, high Physical Activity Levels (PAL), and a rational diet; (3) Individuals exhibiting orthorexic behavior tend to overestimate the portion sizes of products and foods on photo models; and (4) There is a correlation between orthorexic behavior and other eating behaviors.

## 2 Materials and methods

The study is a continuation of the original research project presented in the previous papers published in 2022 in *Nutrients* (42, 43). The present article, by demonstrating new relationships, complements the previously presented fragmented results, thereby expanding current scientific knowledge without raising any doubts regarding the reliability and research ethics (44). Moreover, the results were obtained during the implementation of a large research project, and their full presentation in a single manuscript was realistically impossible.

### 2.1 Sample group

The research encompassed 600 participants, evenly distributed between two academic domains: health-related fields (HRF) and non-health-related fields (NRF). The sample size was determined using the minimum sample size formula (*N*_min_ = [NP(α2·*f*(1 − *f*))]/[NP·*e*2 + α2·*f*(1 − *f*)], where: *N*_min_—minimum sample size; NP—the size of the population from which the sample is taken; α*—*confidence level for the results; *f*—the size of the fraction; *e*—the assumed maximum error), taking into account the total number of students in a specific year at a particular university. This ensured a representative sample group. The survey questionnaire was administered to all students in a specific year and field of study, achieving a response rate of 83.1%. All participants were in the final year of their master’s program, with the HRF group comprising dietetics and physical education students, while the NRF group consisted of management and computer science students. The main variable taken into account was the field of study, not the age of the students. The students came from the following universities: Medical University of Silesia, Academy of Physical Education in Katowice, University of Silesia and University of Economics in Katowice.

The surveyed group is representative of the Polish population, according to the Central Statistical Office; almost 1.2 million citizens are currently studying in Poland. Based on calculations, the minimum sample size for such a population is 384 cases, so the group of 600 people selected for the study meets the minimum research sample. The minimum sample size allows us to calculate how many elements (individuals, re-search units) we should survey so that our results with a certain confidence level and an assumed maximum error estimate the true results in the population.

### 2.2 Eligibility criteria

During the material collection phase, 689 correctly completed questionnaires were recorded, but 89 questionnaires had to be rejected due to restrictive inclusion criteria. The criteria are described below.

The HRF group included 300 students majoring in dietetics and physical education, selected for their specialized knowledge in rational nutrition and physical activity. In contrast, the NRF cohort consisted of 300 final-year students specializing in management and computer science, chosen for their absence of specialized knowledge in the aforementioned domains at the university level. Gender balance was taken into account in the selection of majors, considering the customary gender preferences for specific fields. Data were collected from January to March 2021. All final-year students were eligible. All students studied in Polish (Silesia region).

Exclusions from the NRF group involved individuals with prior or concurrent education in health-related fields, as well as those utilizing knowledge of rational nutrition and physical activity in their professional roles. Respondents with medical conditions affecting diet and physical activity, such as allergies, metabolic diseases, or tumors, were also excluded. Likewise, participants following specific dietary models (e.g., elimination diets) or experiencing pregnancy and puerperium were excluded.

The investigation targeted final-year students because of their anticipated in-depth understanding of health sciences and physical culture sciences, particularly among health science students. The inclusion of final-year students was also extended to the NRF group in order to uphold uniformity in inclusion criteria and guarantee a reasonably homogeneous student group.

In addition, people with a history of mental disorders, other eating disorders and substance abuse were excluded from the study. These data were subject to self-reporting in the survey questionnaire.

The study received approval from the Bioethics Committee of the Medical University of Silesia in Katowice (code: PCN/CBN/0052/KB/127/22, date: July 12, 2022), by the Act on Medical and Dental Professions of December 5, 1996, which defines medical experimentation. Additionally, participants in the study provided informed consent for their involvement.

### 2.3 Research tools

The body mass index (BMI) was calculated using the formula: BMI (kg/m^2^) = body weight (kg)/height (m^2^). The weight and height were self-reported by the surveyed students. The results were then evaluated using the following classifications (16): ≥30.00 kg/m^2^, signifying obesity; 25.00–29.99 kg/m^2^, indicating overweight; 18.50–24.99 kg/m^2^, representing normal body weight; 17.00–18.49 kg/m^2^, suggesting underweight; and ≤ 16.99 kg/m^2^, denoting malnutrition.

The dietary assessment utilized a tool developed by the author based on Polish nutrition standards (45), comprising 20 dietary indices such as consumption frequency of specific food groups, meal count per day, meal regularity, snacking, and fluid intake. Respondents indicated “”yes” or “no” for each nutrition-related question and received one point for every correct answer based on established standards, with a maximum possible score of 20. The results were categorized using the following scale: 18–20 points for very good nutrition, 14–17 points for good nutrition, 10–13 points for moderate nutrition, and ≤ 9 points for poor nutrition. This questionnaire had been used in a prior study by the authors. The tool underwent validation through initial feedback from 10 specialists in the field, with revisions made based on their suggestions. Subsequently, the questionnaire was pilot-tested twice on a group of 30 adults, 2 weeks apart, and π Scott’s coefficient was calculated to measure the questionnaire’s consistency and relevance, yielding a relevance score of 0.93 (very good) for specific questions and 0.72 (good) for others (46).

Based on the physical activity score obtained from the survey, participants were classified into distinct PAL in accordance with established physical activity guidelines: 1.2 for sedentary behavior; 1.4 for low physical activity (approximately 140 min per week); 1.6 for moderate physical activity (around 280 min per week); 1.8 for high physical activity (about 420 min per week); and 2.0 for very high physical activity (around 560 min per week) (47).

The second version of the Exercise Motivations Inventory (EMI-2) questionnaire encompasses 56 variables distributed across 14 categories that delineate exercise motives. Within each category, participants could allocate five points, where 0 denoted the highest priority for the motivator and 5 signified the lowest priority. Consequently, lower scores for a particular motivator suggest a greater degree of motivation toward it (48).

The Düsseldorf Orthorexia Scale (DOS) test comprises 10 questions allowing respondents to choose between “agree’ and “disagree.” It offers a scoring range of 10–40 points. A score between 25 and 29 suggests the presence of orthorexic tendencies, while a score surpassing 30 indicates the presence of orthorexia (27).

The Three-Factor Eating Questionnaire (TFEQ-13) questionnaire facilitates the evaluation of three behaviors through a set of 13 questions distributed across three subscales: five questions pertain to eating restriction (RE) (questions 1, 9, 10, 12, and 13), five questions address a lack of control over eating (LCE) (questions 2, 5–7, and 11), and three questions directly inquire about eating influenced by emotions (EE) (questions 2, 4, and 8). Standardized responses are provided on a four-point scale, ranging from zero to three. Respondents select the most applicable statement for each question from options including “definitely yes,” “rather yes,” “rather no,” and “definitely no.” Scores are tabulated independently for each sub-scale, where a higher score indicates a more pronounced manifestation of the behavior. The overall scale exhibited a satisfactory internal consistency alpha Cronbach’s coefficient of 0.78. For the individual subscales, it was 0.78 for eating restriction, 0.76 for lack of control over eating, and 0.72 for eating under the influence of emotions. Significantly, all subscales displayed positive and statistically significant correlations with one another (*p* = 0.001) (49).

### 2.4 Study procedure

The study involved a survey questionnaire and an album showcasing sample foods and dishes. Ethical guidelines, anonymity principles, and the RODO clause (Polish Law on Respect for Classified Information) were strictly followed during the study. The survey was administered through an online form, an accepted method in psychological research. Participants were sent the questionnaire link via dedicated email accounts. To prevent fake or bot responses, measures were taken during data collection, including checking login and completion times. The questionnaire was further secured with a CAPTCHA key.

In the second phase of the study, participants were presented with an album featuring sample foods and dishes. This album aimed to evaluate their ability to estimate portion size and calorie content. It comprised 12 photographs, each representing one of the 12 food groups (50). Before each study, participants’ visual perception (image interpretation) was assessed using select Ishihara and optical illusion boards. These tools are commonly used to evaluate visual color perception and object perception in images (e.g., size, shape, and length). To link questionnaire results with the album, each participant was assigned a unique number while completing the questionnaire, which was then used to identify their responses in the album.

### 2.5 Statistical analysis

Data obtained from the survey questionnaire were organized into tables for descriptive examination, involving the calculation of percentages (%), counts (*N*; *n*), mean (*X*), and standard deviations (SD). Extensive statistical analyses were conducted to evaluate discrepancies between the exhibited behaviors (pro-health or anti-health) and the occurrence of EE within the sample cohort. The statistical analysis involved the utilization of chi-square (χ^2^), U Mann–Whitney, and Kruskal-Wallis ANOVA tests. Kruskal-Wallis ANOVA test (Kruskal-Wallis H test), is used to compare three or more independent groups in terms of median or other central position. It is a non-parametric alternative to the one-way ANOVA and is used when the normality of the data distribution cannot be assumed. The odds ratio was calculated based on pooled calculations. The numbers of successes were divided by the number of failures in each group, and then the score of one group was divided by the score of the other group. The study adopted a pre-established significance level of *p* = 0.05.

## 3 Results

In the HRF group, women accounted for 55%, men 45%. In the NRF group, women accounted for 58%, men 42%. The statistical test showed no differences between the subjects in terms of the gender trait (*p* < 0.05). The social diversity of the subjects was present, but it was not significant and did not affected the final results of the study (*p* < 0.05). All respondents were students in the final year of their Master’s degree. Nearly 90% of the respondents lived in cities with a population of more than 100,000. More than 80% of the respondents did not work permanently in any place. The mode of study and education of the respondents’ parents were not reportable.

Considering the most significant motivating factors for physical activity, it was demonstrated that within the examined group, important motivators included (Table 1).

**Table 1:** The most significant motivating factors for physical activity.

Motivating factors for physical activity	Mean and SD (points)^*^	HRF	NRF	*T*	*f*	*p*
Disease prevention	2.08 ± 1.75	2.08 ± 1.71	2.08 ± 1.74	-	-	-
Maintaining health	2.06 ± 1.85	2.34 ± 1.75	1.80 ± 1.74	15.381	0.582	0.001
Strength/endurance	2.03 ± 1.84	2.02 ± 1.75	2.04 ± 1.79	-	-	-
Physical appearance	1.83 ± 1.86	182 ± 1.72	184 ± 1.80	-	-	-
Mental rejuvenation	1.85 ± 1.77	186 ± 1.78	184 ± 1.71	-	-	-
Weight control	1.77 ± 1.91	1.77 ± 1.76	1.81 ± 1.82	17.002	0.489	0.001
Exercise enjoyment	1.79 ± 1.84	178 ± 1.81	180 ± 1.84	-	-	-

^*^0—highest priority; 5—lowest priority.

The intensity of specific motivations significantly varied between the HRF and NRF groups. The mean rank in the “maintaining health” subscale for HRF was (2.34 ± 1.75); significantly lower than the mean rank for NRF, which was (1.80 ± 1.74). This indicates that maintaining health is the strongest incentive for physical activity among HRF. In the ‘weight control’ subscale, the mean rank for HRF was (1.77 ± 1.76), higher than the mean rank for NRF (1.81 ± 1.82). Therefore, weight control is the most significant motivator for exercise in the NRF group. Both noted relationships were statistically significant (*p* = 0.001). Additionally, a statistically significant relationship was observed among individuals with low BMI values (98.2% of the underweight group and 65.7% of the normal weight group), and the motivator ‘disease prevention’ was revealed to be significant (*p* = 0.001). In this case, no differences were found between the study groups.

None of the participants examined were identified as malnourished. Approximately 15.5% of the participants were underweight. The majority of participants, about 61.6%, had a normal weight. Overweight and obesity were predominantly found in the NRF group, making up 23.7%. Statistically significant differences were observed concerning the prevalence of overweight and obesity in the NRF group (*p* = 0.001). In terms of dietary assessment, the HRF group demonstrated the most favorable dietary pattern, with 97.5% of students displaying a very good or good dietary pattern (84.0 and 13.5%, respectively). Conversely, the NRF group predominantly exhibited a moderate dietary pattern, constituting 64.7% of all cases in this group. A less common dietary pattern classified as “good” was identified in only 24.9% of cases within the NRF group. It is worth noting that an inadequate dietary pattern was predominantly observed in individuals from the NRF group (3.6% of the total participants).

Analyzing the data on physical activity and the motivations connected to it, it was noted that the majority of the HRF group (98.9%) and a significant portion of the NRF group (83.3%) demonstrated varying degrees of physical activity. Nearly half of the participants (46.5%) exhibited a low level of physical activity, with a notable prevalence among the NRF group (79.2%). In terms of moderate physical activity, approximately a quarter of the participants (25.4%) fell into this category, distributed across both the HRF group (33.5%) and the NRF group (16.7%). On the other hand, high to very high levels of physical activity were reported by over a quarter of the students (28.4%), with a substantial proportion coming from the HRF group (48.1%).

During the evaluation of portion sizes, it was noted that 32.7% of participants overestimated the sizes of products and dishes depicted in the images. The majority of this demographic belonged to the HRF group, accounting for 57.9%, whereas a smaller proportion came from other fields of study, totaling 7.2%. Conversely, in the case of underestimation (34.1%), primarily individuals from the NRF group underestimated the sizes of the products and dishes, comprising 56.5%. In contrast, within the HRF group, the percentage of underestimations was notably lower (10.8%). The remaining participants accurately assessed the portion sizes, accounting for 34.1%. After a slight adjustment of 0.3%, the overestimation increased to 33%, while the underestimation decreased to 34.5%. Upon analyzing the outcomes of the test regarding the capacity to estimate the caloric content of portions based on images, it was observed that 36.1% of participants overestimated the caloric content of the products and dishes presented in the images. Predominantly, individuals affiliated with health disciplines constituted this group (58.6%), whereas those without such affiliations were less prevalent (13.3%). On the contrary, in the case of underestimating the caloric content of the dishes (35.5%), individuals from the NRF group most frequently underestimated the energy value of the products and dishes featured in the album (55.8%); within the HRF group, such instances were considerably less common (15.6%). The remaining participants accurately indicated the caloric content of the portions, accounting for 29%. Following a minor adjustment of 0.3%, the percentage of overestimation rose to 36.4%, while underestimation decreased to 35.2%.

The analysis of the DOS questionnaire outcomes revealed that 52.7% of all participants exhibited inclinations toward orthorexia, with 12.3% displaying pathological (full-scale) orthorexia (totaling 390 individuals with orthorexia—65.0% from HRF and NRF group together). Orthorexic tendencies were more prominent in the HRF group compared to the NRF group (Table 2). Based on odds ratio calculations, it was approximated that orthorexia occurs 4.5 times more frequently in the HRF group than in the NRF group. When comparing BMI risk groups for orthorexia, it was observed that individuals with orthorexic tendencies from the HRF group had lower BMI values (*p* = 0.002). Similarly, in terms of diet, individuals with good and very good nutritional habits were more likely to be associated with the group at higher risk of orthorexia. In this instance, a statistically significant correlation with HRF group membership was also established (*p* = 0.001). Through the conducted statistical inference, it was deter-mined that high PAL values were prevalent in the HRF group exhibiting orthorexic tendencies, indicating a statistically significant association between the variables (*p* = 0.002). Regarding motivations, it was revealed that the primary driver for engaging in physical activity among the group displaying orthorexic tendencies was health concern. These motivations were selected three times more frequently by individuals from the HRF group than from the NRF group (*p* = 0.001). Furthermore, a statistically significant relationship was demonstrated in both the estimation of portion sizes and caloric content of meals; individuals from the HRF group with orthorexic behaviors tended to overestimate portion sizes and caloric content in the photographic model test (*p* = 0.002).

**Table 2:** Identified risk factors for orthorexia in HRF I NRF groups (*n* = 390).^*^

Variable	Orthorectic HRF	Orthorectic NRF	*T*	*f*	*p*
BMI < 25 kg/m^2^	59.7% (233)	40.3% (157)	13.644	0.562	0.002
Good diet quality	54.4% (212)	45.6% (178)	11.443	0.633	0.001
PAL > 1.6	55.2% (215)	44.8% (175)	14.002	0.459	0.002
Health-related physical activity motivation	51.2% (200)	48.8% (190)	10.982	0.458	0.001
Overestimated portion size	55.9% (218)	44.1% (172)	11.323	0.781	0.001
Overestimated caloric size	58.7 (229)	41.3% (161)	12.034	0.433	0.002

N, Sample size; HRF, Health-related fields; NRF, Non-health-related fields. ^*^The results presented are only for the percentage of the risk factor in the subgroup studied.

Based on TFEQ-13 scores, 33.4% exhibited dietary restriction (RE) behaviors (HRF: 62.6%; NRF: 10.1%), while lack of control over food intake (LCE) was exhibited by: HRF—11.7%; NRF—15.2%. Emotional eating (EE) was present in 57.7%, with the NRF group being the main demographic group (73.5%). There was no correlation between LCE and EE and the incidence of orthorexia in the study group (*p* > 0.05). However, a correlation was observed between the incidence of orthorexia and RE (*p* = 0.001). In this group, 85.5% of orthorexia cases also exhibited RE, and the condition was 2.5 times more common in the ON group (Table 3).

**Table 3:** Relationship between prevalence of orthorexia and selected eating behaviors (*n* = 390).

TFEQ-13	HRF	NRF	*T*	*f*	*p*
	*n* = 390	*n* = 210			
Restrictive eating (RE)	62.6% (244)	10.1% (39)	10.091	0.289	0.001
Lack of control over eating (LCE)	11.7% (46)	15.2% (59)	–	–	–
Emotional eating (EE)	25.7% (100)	73.5% (286)	–	–	–

*n*, Orthorectic sample size; HRF, Health-related fields; NRF, Non-health-related fields.

## 4 Discussion

The research conducted within the framework of the study on orthorexic behavior and emotional eating among young people related to the issue of taking care of health and fitness allowed us to learn about the determinants of the occurrence of the indicated phenomena. Primarily, it has been confirmed that individuals exhibiting behaviors deemed health-promoting are characterized by a greater risk of developing orthorexia. These same individuals tend to overestimate the size and caloric content of foods and dishes on photo models presented to them. In addition, no links have been found between orthorexic behavior and emotional eating. However, orthorectic tendencies are associated with a behavior referred to in the literature as restrictive eating. In instances of emotional eating, it was observed to be more prevalent among individuals characterized by insalubrious and sedentary lifestyles, manifested through diminished levels of physical activity and erratic eating patterns. The same people in the study on estimating portion sizes and calories based on photographs showed a tendency to underestimate the portions and food items visible in them.

Learning about the motivators for physical activity in groups at potential risk for non-specific eating disorders seems reasonable, as they can provide an early diagnostic signal (51, 52). In the study provided by López-Gil et al. (53), data concerning the prevalence of orthorexia nervosa symptoms across 18 countries were presented, involving a total sample of over 30,000 participants. This meta-analysis reveals that the overall proportion of orthorexia nervosa symptoms stands at 27.5%, with marginal differences observed between genders. The highest prevalence of orthorexia nervosa symptoms was observed among individuals focused on sports performance or body composition. Furthermore, the analysis indicates a rising trend in the prevalence of these symptoms over the years, highlighting a significant public health concern and underscoring the necessity for the development of psychometric tools to support clinical diagnosis and treatment efficacy. In Poland, the situation regarding orthorexia is also noteworthy. Emerging research suggests a growing awareness of orthorexia nervosa within the country’s population, however, with an increasing number of cases reported in recent years (42, 43). This underscores the importance of further investigation and intervention strategies tailored to the Polish context.

Several studies cited earlier noted that groups of individuals at risk for orthorexia have an above-average interest in the topic of maintaining a healthy life-style (13, 54–57). Such a group is very likely to include health science students (16, 28, 37). It is noted that in orthorexics, the motivation may or may not be weight loss, and the main driving force behind the disorder is the need to maintain health. It is emphasized that the primary motivator in undertaking activities aimed at improving and maintaining health in orthorexics will be reasons related to physical health, its regulation and maintenance (51). This includes motives for engaging in physical activity, which is a direct factor in maintaining health (52).

There are scientific reasons to suspect that behaviors indicative of orthorexia are more common in groups of people concerned with health and proper body shape (33, 36, 37). Several research studies emphasize the link between orthorexia and physical activity. For example, Herranz et al. (58) observed a higher prevalence of orthorexia in a group of yoga practitioners. Segura-García et al. (59) showed that in a group of athletes studied, 60% showed orthorexic tendencies. Similar relationships have been shown in groups of dancers, runners, swimmers, cyclists, and gym-goers (13, 55–57). This is accompanied by the creation of certain beliefs and patterns of compulsive repetition of certain activities. Such schematic behavior is often associated with the occurrence of anankastic personality (a.k.a. obsessive-compulsive personality) (OCPD) (60), a personality disorder during which a person feels an internal compulsion to act according to strictly defined procedures—the is not flexible and acts ac-cording to a scheme: “black or white”; “all or nothing” (61, 62).

Exercise is not only associated with improving fitness and functioning of the body but also with regulating emotions, which may be an important aspect of orthorexics (63, 64). Because orthorectic behavior can be a response to inappropriate emotion regulation, which in turn may contribute to the development of faulty eating patterns. Such as emotional overeating or restricting food intake, as well as misperception of food size and caloric content (65, 66). In their study, Strahler et al. highlighted that individuals with orthorexia exhibit markedly elevated levels of stress, depression, and anxiety, along with diminished life satisfaction when compared to their healthy counterparts (67). In other studies, orthorexia tendencies have been linked to other eating behaviors such as restricting food intake or emotional eating (68–70). In the aforementioned study (68), female students with orthorexia claimed that negative emotions lead them to increase cravings or restrict food intake. Similar emotional-motivational relationships were found in studies by Koven and Senbonmatsu (71) and Tan and Chow (69).

The scientific literature distinguishes between two emotion-based phenomena that project into the realm of eating behavior: emotional eating and food restriction (69). Both phenomena can be linked to stress and the occurrence of strong emotions in life. In the case of emotional eating, the main problem is that affected individuals con-fuse psychological signals with physiological ones, in effect consuming food at a time when real hunger is not present (70). Eating restriction can also be based on situations associated with elevated levels of stress, and the effect of this phenomenon is restraint in food intake or restriction of certain foods (72). Research to date emphasizes that normal-weight individuals may exhibit behaviors typical of emotional eating, but it is more of a problem for overweight individuals (73–75). More than half (57.3%) of overweight or obese adults report high levels of emotional eating (76). Emotional eating alone increases the risk of being overweight 14-fold (77, 78). Additionally, a study by Kowalkowska and Poinhos (79) noted that emotional eating tended to be more prevalent among women, whereas restrictive eating was more frequently observed in men. Another study, which involved 529 adult participants and 358 adolescents, discovered that girls exhibiting higher scores in restrained eating consumed fewer calories than their counterparts, while those characterized as emotional eaters had a greater intake of snack foods (80). According to results, emotional eating and uncontrolled eating are positively correlated in both genders, with the relationship being stronger in females (79, 81). The literature further emphasizes that orthorexia itself may be a response to negative emotions experienced by creating a way of coping (82–85). Hence, the connections between these phenomena may be legitimate.

## 5 Strengths and limitations

At this point, it is worth emphasizing that an important strength of the conducted research is that, in light of current scientific reports, the material developed appears to be a novel approach to non-specific eating disorders. Few studies in the scientific literature approach the topic from a psychodietetic angle—this approach allows a comprehensive assessment of the phenomenon and the setting of further research goals. The main goal of the conducted research was to identify the phenomenon of non-specific eating disorders among a group of people working in the field of health (dietetics and physical education). Of course, it was not assumed that it is the field of study that defines the type of unfavorable eating behavior, but rather certain personality conditions of the individual who chooses a particular field of study. It is worth emphasizing here that this aspect should be expanded with further research.

It should be noted that the research methods used have some limitations, but also development potential. Thus, in the future, it would be advisable to expand the scope of the research to include other social groups, and a larger number of research tools and deepen the existing conclusions. The main limitation of the research was that it was conducted using a diagnostic survey method with the help of online forms. The adoption of such a procedure was justified by the fact that the research part fell in 2020–2023 when the COVID-19 pandemic prevailed in the world. In order to mitigate potential response biases and minimize the risk of distortions, various precautions were implemented, including the use of access keys (CAPTCHA) and monitoring login times. In addition, the safeguards used completely reduced the risk of a fake/bot responder. An additional limitation was that the identification of respondents’ behavior was based only on psychometric tools, so to minimize possible error the tools used were validated. Additionally, a limitation of the study can be considered the use of self-reported data regarding height and weight, which could be subjective and thus occasionally inaccurate. Another limitation of the study is that no other tools were used to study eating disorders, which will be expanded upon in future studies. Furthermore, due to the homogeneity of the population, the study did not examine the effect of gender and demographic characteristics on the prevalence of orthorexia. These will be undertaken in future research projects.

## 6 Practical implications

This study is important due to the lack of official recognition of orthorexia as an eating disorder and the associated behaviors, which may constitute predisposing factors to the development of eating disorders. By assessing the frequency of orthorexia among students with different lifestyles, including dietary habits and levels of physical activity, as well as their motivations, this study provides significant information. It identifies high-risk groups, especially students in health-related fields, who due to their strong health awareness may be more susceptible to orthorexic behaviors. Analyzing the motivations behind dietary habits and physical activity helps understand factors influencing orthorexic tendencies. The results indicate that these tendencies may be associated with lower BMI, better diet quality, and higher levels of physical activity, suggesting significant health consequences.

Simultaneously, while the studies conducted as part of this project are epidemiological in nature and contribute significantly to theories regarding eating disorders, it is important to emphasize their contribution to practice and potential future actions in the field of health promotion. Primarily, these studies managed to identify phenomena related to unfavorable dietary behaviors among young people. Therefore, further actions should focus on promotional and educational activities in identified high-risk groups. Given that the goals of national health promotion strategies include actions aimed at reducing the effects of mental disorders, including eating disorders, in society, as well as psychoeducation for high-risk groups. The conclusions drawn from the study provide an important lesson in further planning and implementing health policy programs and building support groups for individuals with problematic eating behaviors. Additionally, given the nature of the studied groups, it is worth considering incorporating into research programs sessions on methods and techniques for coping with stress and emotional regulation, as well as promoting basic psychological self-help skills.

## 7 Conclusion

For young individuals pursuing studies in health-related fields, their motivation to engage in regular exercise is primarily driven by a keen awareness of health issues. This heightened consciousness of health, however, can sometimes lead to a concerning phenomenon known as orthorexia. Orthorexia is an obsessive focus on consuming a “perfect” or excessively healthy diet, which can have detrimental effects on one’s over-all well-being. Interestingly, those involved in health care topics or professions, who already adhere to what is commonly considered a healthy lifestyle—comprising regular physical activity and a nutritious diet—are at an increased risk of developing orthorexic tendencies. This underscores how the pursuit of health and knowledge in health-related domains can inadvertently foster a harmful fixation on dietary habits and body image. Individuals with orthorexic tendencies often exhibit a distorted perception of food portions and calorie content, perceiving them as larger than they truly are. This skewed perception reflects the rigidity and obsession associated with orthorexia, further emphasizing the need for awareness and intervention. Moreover, research has revealed a correlation between orthorexic behavior and food restriction. Those who engage in food restriction practices, whether due to dietary beliefs or body image concerns, are more susceptible to developing orthorexic tendencies. This inter-connection underscores the complex and multifaceted nature of disordered eating patterns and the importance of addressing both physical and psychological aspects of health in the education and support provided to young individuals in health-related fields.

In conclusion, it is worth emphasizing that orthorexic behavior *per se* is very often indicative of leading a pro-health lifestyle, which at first glance is not so bad, but as a consequence, worsening orthorexia can lead to many pathologies, such as the development of other eating disorders, such as anorexia or bulimia. In view of this, the results of the cited study may be a contribution to deepening expertise related to the prevalence of orthorexia in the general population and the study of its relationship with other eating disorders.

### 7.1 Implementation

Research findings on orthorexia among young individuals provide valuable insights for practitioners and clinicians to undertake effective actions in both mental and physical health domains. Based on these studies, specific strategies can be devised to aid in the identification, prevention, and treatment of this disorder. Firstly, community education about orthorexia is crucial. By organizing workshops, lectures, and seminars, awareness about this disorder can be raised among both young individuals and their caregivers. Secondly, routine screening in clinical practice can help swiftly identify individuals at risk of orthorexia. Diagnostic tools based on research findings can assist doctors and therapists in diagnosing this disorder. Thirdly, therapeutic interventions based on research data, such as cognitive-behavioral therapy, can be effective in treating orthorexia. Developing therapeutic programs tailored to the specific needs of young individuals is paramount. Fourthly, promoting healthy eating habits without falling into obsessive behaviors is essential. Practitioners and clinicians can use research findings to develop dietary guidelines emphasizing moderation, a balanced approach to eating, and enjoyment of meals. Fifthly, collaboration among specialists such as dietitians, psychiatrists, and family physicians is crucial in providing comprehensive care for individuals affected by orthorexia. Collaborative teamwork can offer more effective support for patients. Finally, further research on orthorexia is imperative. Such research could focus on identifying risk factors, the effectiveness of various therapeutic interventions, and the influence of social media and culture on the development of this disorder. In summary, research findings on orthorexia among young individuals serve as a foundation for practical actions for practitioners and clinicians. These findings enable effective responses to this disorder through identification, prevention, and treatment, contributing to the improvement of mental and physical health among the younger generation.

## Data availability statement

The original contributions presented in the study are included in the article/supplementary material; further inquiries can be directed to the corresponding author.

## Ethics statement

The studies involving humans were approved by Bioethics Committee of the Medical University of Silesia in Katowice. The studies were conducted in accordance with the local legislation and institutional requirements. The participants provided their written informed consent to participate in this study. Written informed consent was obtained from the individual(s) for the publication of any potentially identifiable images or data included in this article.

## Author contributions

MR: Formal analysis, Project administration, Resources, Software, Supervision, Visualization, Writing – original draft, Writing – review & editing. MG: Conceptualization, Data curation, Investigation, Methodology, Resources, Writing – original draft, Writing – review & editing. KK-K: Investigation, Methodology, Writing – review & editing, Writing – original draft. EM-M: Formal analysis, Resources, Supervision, Writing – original draft, Writing – review & editing.
